# Supplementary material for: Large genome-wide association study identifies three novel risk variants for restless legs syndrome
Source: Commun Biol. 2020 Nov 25;3:703. doi: 10.1038/s42003-020-01430-1 (PMC7689502; doi:10.1038/s42003-020-01430-1)
Supplement: Supplementary file 1 — Supplementary Information [file 42003_2020_1430_MOESM1_ESM.pdf]

SUPPLEMENTARY MATERIAL

Title of the paper:

Large genome-wide-association study identifies three novel risk variants for restless legs syndrome

Authors:

Maria Didriksen*[1,2], Muhammad Sulaman Nawaz*[2,3], Joseph Dowsett[1], Steven Bell[4,5,6], Christian Erikstrup[7], Ole B. Pedersen[8], Erik Sørensen[1], Poul J. Jennum[9,10], Kristoffer S. Burgdorf[1], Brendan Burchell[11], Adam S. Butterworth[4,5,6], Nicole Soranzo[11,12], David B. Rye[13], Lynn Marie Trotti[13], Prabhjyot Saini[13], Lilja Stefansdottir[2], Sigurdur H. Magnusson[2], Gudmar Thorleifsson[2], Thordur Sigmundsson[3,14], Albert P. Sigurdsson[3], Katja Van Den Hurk[15], Franke Quee[15], Michael W. T. Tanck[16], Willem H. Ouwehand[4,12,17], David J. Roberts[18,19], Eric J. Earley[20], Michael P. Busch[21], Alan E. Mast[22], Grier P. Page[23], John Danesh[4,5,6,17], Emanuele Di Angelantonio[4,5,6], Hreinn Stefansson[2], Henrik Ullum[1**], Kari Stefansson[2**]

[1]Department of Clinical Immunology, Copenhagen University Hospital, Rigshospitalet, Copenhagen 2100, Denmark.

[2]deCODE Genetics, Reykjavik 101, Iceland.

[3]Faculty of Medicine, University of Iceland, Reykjavik, 101, Iceland.

[4]The National Institute for Health Research Blood and Transplant Unit in Donor Health and Genomics, University of Cambridge, Cambridge CB1 8RN, UK.

[5]UK Medical Research Council/British Heart Foundation Cardiovascular Epidemiology Unit, Department of Public Health and Primary Care, University of Cambridge, Cambridge CB1 8RN, UK.

[6]British Heart Foundation Centre of Excellence, Division of Cardiovascular Medicine, Addenbrooke's

Hospital, Cambridge CB2 0QQ, UK.

[7]Department of Clinical Immunology, Aarhus University Hospital, Aarhus, Denmark.

[8]Department of Clinical Immunology, Nastved Sygehus, Nastved, Denmark.

[9]Department of Clinical Neurophysiology, Danish Center for Sleep Medicine, Copenhagen University Hospital, Rigshospitalet, Glostrup, Denmark.

[10]Faculty of Health, University of Copenhagen, Copenhagen, Denmark

[11]Faculty of Human, Social and Political Sciences, University of Cambridge CB1 8RN, Cambridge, UK

[12]Department of Haematology, University of Cambridge, Cambridge Biomedical Campus, Cambridge CB2 0PT, UK.

[13]Department of Neurology and Program in Sleep, Emory University, Atlanta, GA

[14]Department of Psychiatry, Telemark Hospital Trust, Skien, Norway

[15]Department of Donor Studies, Sanquin Research, Amsterdam 1066CX, Netherlands

[16]Department of Clinical Epidemiology, Biostatistics and Bioinformatics, Amsterdam UMC, University of Amsterdam. Amsterdam, The Netherlands

[17]Department of Human Genetics, The Wellcome Trust Sanger Institute, Wellcome Trust Genome Campus, Hinxton, Cambridge CB10 1HH, UK.

[18]National Health Service (NHS) Blood and Transplant and Radcliffe Department of Medicine, NIHR Oxford Biomedical Research Centre, University of Oxford, John Radcliffe Hospital, Oxford, UK:

[19]BRC Haematology Theme and Department of Haematology, Churchill Hospital, Oxford, UK.

[20]RTI International, Research Triangle Park, North Carolina, USA.

[21]Vitalant Research Institute, San Francisco, California, USA, and Department of Laboratory Medicine, University of San Francisco, San Francisco, California, USA.

[22]Blood Research Institute, Versiti, Milwaukee, WI, USA.

[23]RTI International, Atlanta, Georgia, USA.

* These authors contributed equally; ** These authors jointly supervised this work

CONTENT

Supplementary methods 4

Cohorts included in the discovery meta-analysis 4

Questionnaires used to assess RLS 6

Cohorts used for follow-up/replication analysis 8

EU-RLS-GENE study 8

US (RBC-Omics) cohort 8

Genotyping, imputation, and association analysis of cohorts included in the discovery meta-analysis 10

Icelandic dataset 10

Danish (The Danish Blood Donor Study) dataset 10

US (Emory) dataset 11

UK (The INTERVAL Study) dataset 12

The UK (UK Biobank) dataset 13

The Netherlands (Donor InSight-III) dataset 13

Meta-analysis 14

Genotyping, imputation, and association analysis of cohorts used for follow-up/replication analysis 15

EU-RLS-GENE consortium dataset 15

US (RBC-Omics) dataset 16

Genetic risk analyses: UK Biobank 24

Supplementary references 31

Supplementary methods

Cohorts included in the discovery meta-analysis

In total, 480,982 (10,257 cases and 470,725 controls) Caucasians of European ancestry (from Iceland, Denmark, the UK, Netherlands, and the US) were recruited for the study (Figure 1). The Icelandic dataset comprised individuals who responded to a media inquiry for those with RLS-like symptoms and subjects that participated in various studies at deCODE Genetics and who had answered a screening questionnaire. RLS was assessed by using a self-completed questionnaire based on the International RLS Study Group diagnostic criteria[1] (IRLSSG). All participating individuals who donated blood provided written informed consent. All sample identifiers were encrypted in accordance with the regulations of the Icelandic Data Protection Authority. Approval for the study was provided by the National Bioethics Committee of Iceland.

Both the DBDS and the INTERVAL subjects were recruited from blood banks in Denmark and in the UK, respectively. RLS status among the blood donors was assessed using the Cambridge-Hopkins RLS questionnaire (CH-RLSq), which is a 10-item questionnaire with excellent diagnostic specificity (94%) and sensitivity (87.2%), albeit somewhat inflated due to their being derived from a specialized blood donor cohort validated against telephone interview versus face-to-face clinical impression and informed by PLM determinations in a general population [2]. Definite and probable RLS cases were combined into one group and the remaining participants were included in analyses as controls. All DBDS participants provided written informed consent. The DBDS dataset was approved by The Scientific

Ethical Committee of Central Denmark (M-20090237) and by the Danish Data Protection agency (30-0444). GWAS studies in DBDS were approved by the National Ethical Committee (NVK-1700407). The INTERVAL dataset was approved by the National Research Ethics Service Committee East of England - Cambridge East (Research Ethics Committee (REC: 11/EE/0538). Written informed consent was collected from all INTERVAL participants.

For UK Biobank participants, the clinical diagnostic code International Classification of Diseases, tenth revision: G25.8 (ICD10) was used to inform about case status of restless leg syndrome. The specific sub-code for RLS (G25.81) was not available. UK Biobank is approved by the North West Multi-centre Research Ethics Committee, and by the Patient Information advisory Group, the National Information Governance Board for Health and Social Care, and from the Community Health Index Advisory Group. UK Biobank also holds a Human Tissue Authority license.

Furthermore, we included a dataset from the Program in Sleep at Emory University, which is a tertiary care center for RLS that is recognized as a Quality Care Center for RLS by the RLS Foundation. RLS affectation status in this dataset was limited to adults of Northern European origin (i.e., African Americans, Hispanics and Asians were excluded). RLS status was assessed by one of two clinicians familiar with RLS (David B. Rye and Lynn Marie Trotti) complemented by objective measurements of periodic leg movements in sleep (PLMS) and additional secondary and supportive diagnostic features[1]. Thus, RLS status was clinically verified. An institutional review board at Emory University, Atlanta, Georgia, US, approved the study protocol (HIC ID 133-98). All participants provided written informed

consent. The Donor InSight-III (DIS-III, 2015-2016) is the second follow-up of an observational cohort study conducted among blood and plasma donors in the Netherlands aimed to gain insight into characteristics, health and behavior of donors[3]. RLS status was self-reported using the questionnaire developed as part of the RISE study[4]. The questionnaire is based on the IRLSSG criteria and it was developed by REDS investigators in collaboration with an expert on RLS (Professor David B. Rye)[4]. The Medical Ethical Committee of the Academic Medical Center (AMC) in the Netherlands, and Sanquin's Ethical Advisory Board approved DIS-III and all participants gave their written informed consent.

The effect estimates for the 20 known RLS-associated variants[5] were largely similar to the effect estimates observed in each of the cohorts included in this meta-analysis (Supplementary Table 2). This indicates that the phenotypes in each cohort are comparable to previous RLS GWAS efforts. Moreover, the meta-analysis of the discovery and follow-up samples replicated 19 of the 20 previously reported variants.

Questionnaires used to assess RLS

IRLSG RLS diagnostic criteria[1]	CH-RLSq[2]	Questionnaire used by the InSight-III cohort[4]
1. An urge to move the legs usually but not always accompanied by or felt to be caused by uncomfortable and unpleasant sensations in the legs	Do you have, or have you had, recurrent uncomfortable feelings or sensations in your legs while you are sitting or lying down?<br>a) Yes<br>b) No	When you try to relax in the evening or sleep at night, how often do you have unpleasant, restless feelings in your legs that can be relieved by walking or movement?<br>a) Never<br>b) Rarely (2 to 4 times a month)<br>c) Often (5 to 15 times a month)<br>d) Very often (16 or more times a month)
2. The urge to move the legs and any accompanying unpleasant	Do you, or have you had, a recurrent need or urge to move	How often do you experience a strong urge to move your legs

sensations begin or worsen during periods of rest or inactivity such as lying down or sitting.	your legs while you were sitting or lying down?<br><br>a) Yes<br>b) No	usually accompanied or caused by unpleasant sensations in your legs – for example restlessness, creepy-crawly, or tingly feelings?<br><br>a) Never<br>b) Rarely (2 to 4 times a month)<br>c) Often (5 to 15 times a month)<br>d) Very often (16 or more times a month)
3. The urge to move the legs and any accompanying unpleasant sensations are partially or totally relieved by movement, such as walking or stretching, at least as long as the activity continues.	If you get up or move around when you have these feelings do these feelings get any better while you actually keep moving?<br><br>a) Yes<br>b) No<br>c) Don't know	Is the urge to move your legs or are the unpleasant sensations partially or totally relieved by movement such as walking or stretching?<br><br>a) Yes<br>b) No<br>c) Don't know
4. The urge to move the legs and any accompanying unpleasant sensations during rest or inactivity only occur or are worse in the evening or night than during the day.	Are you more likely to have these feelings when you are resting (either sitting or lying down) or when you are physically active?<br><br>a) Resting<br>b) Active	Does the urge to move your legs begin, or do the unpleasant sensations begin or worsen, during periods of rest or inactivity such as when sitting or lying down?<br><br>a) Yes<br>b) No<br>c) Don't know
5. The occurrence of the above features are not solely accounted for as symptoms primary to another medical or a behavioral condition (e.g., myalgia, venous stasis, leg edema, arthritis, leg cramps, positional discomfort, habitual foot tapping).	Which times of day are these feelings in your legs *most* likely to occur?<br><br>a) Morning<br>b) Mid-day<br>c) Afternoon<br>d) Evening<br>e) Night<br>f) About equal at all times	At what times is the urge to move your legs or the unpleasant sensations most bothersome?<br><br>a) In the morning (before noon)<br>b) In the afternoon (before supper)<br>c) In the evening (after supper)<br>d) At night while sleeping<br>e) No difference by time of day
	Will simply changing leg position by itself *once* without continuing to move usually relieve these feelings?<br>a) Usually relieves<br>b) Does *not* usually relieve<br>c) Don't know	

	Are these feelings *ever* due to muscle cramps?<br>a) Yes<br>b) No<br>c) Don't know	
	If so, are they *always* due to muscle cramps?<br>a) Yes<br>b) No<br>c) Don't know	

Cohorts used for follow-up/replication analysis

After the discovery meta-analysis, the novel markers identified in the discovery meta-analysis were tested for replication in two cohorts.

EU-RLS-GENE study

RLS cases in the EU-RLS-GENE study were recruited in specialized outpatient clinics for movement disorders as well as in sleep clinics in eight European countries, French Canada, and the United States. RLS diagnosis was based on a face-to-face interview by an expert neurologist, implementing the diagnostic criteria established by the IRLSSG in 2003. Ancestry-matched controls were obtained for each case sample. A total of 6,228 cases and 10,992 controls was included in the statistical analysis. Written informed consent was obtained from all participants.

US (RBC-Omics) cohort

The RBC-Omics cohort included blood donors recruited from four blood centers in the United States as a part of the Recipient Epidemiology and Donor Evaluation Study (REDS-III)[6-8]. RLS status was assessed using the CH-RLSq as in the DBDS and INTERVAL cohorts.

Analysis in the cohort was restricted to ancestry defined Caucasians and included 423 cases and 7,334 controls. All subjects provided written informed consent.

Genotyping, imputation, and association analysis of cohorts included in the discovery meta-analysis

Icelandic dataset

DNA samples from 150,656 subjects of Icelandic descent were genotyped at deCODE genetics, on one or more of 16 different Illumina SNP genotyping-arrays (including 14,084 subjects as part of this study, 2,636 cases and 11,448 controls). Approximately 34.2 million sequence variants identified through whole-genome sequencing (WGS) (with mean sequencing depth of 10X, median 32X) of 8,453 Icelanders using Illumina GAIIx and HiSeq2000 instruments, were imputed into the 150,656 genotyped Icelanders using the LRP algorithm[9] which yielded LRP haplotypes having high density SNP information (described in detail earlier[10]). Association analysis for each marker was done using logistic regression accounting for cryptic relatedness and adjusting for sex and year of birth[10].

Danish (The Danish Blood Donor Study) dataset

DNA samples from 26,565 subjects from DBDS were chip-typed using the Infinitum Global Screening Array on Illumina® genotyping platform at deCODE Genetics, Iceland. These arrays use ~660,000 common genetic markers which span the entire genome and are representative of all major populations to maximize imputation accuracy. Samples and variants with less than 98% yield were excluded. These samples were long-range phased through Eagle2[11] by using deCODE's North West European reference panel. This panel includes whole genome sequencing data of 15,576 individuals from Scandinavia, the Netherlands and Ireland, 8,429 Danes (1,590 of these are from DBDS). For this reference panel, the Graphtyper [12] variant caller was used to call genotypes from whole genome sequencing data. The chip-genotyping, whole genome sequencing, quality control, long

range phasing, imputation, and association analysis processes were performed at deCODE genetics using the methods described in the section above[10].

US (Emory) dataset

Genotyping was performed at deCODE Genetics, Reykjavik, Iceland, using Illumina Omni Express chips. In QC steps, variants were excluded if they (i) had<94% yield, (ii) had<0.1% minor allele frequency (MAF), (iii) failed Hardy-Weinberg test ($P<1 \times 10^{-6}$) or (iv) showed significant ($P<1 \times 10^{-6}$) difference between genotype batches. Samples with<94% yield were excluded. SHAPEIT (v2.790)[13] was used to phase the resulting genotypes, followed by IMPUTE2 (v2.3.2)[14] to impute un-genotyped variants. To study the population structure and the ancestry of samples we used the ADMIXTURE (v 1.2)[15] and EIGENSOFT (v 6.0.1) software[16]. Samples were excluded if they were identified as ethnic outliers. We used the principal component analysis to identify and exclude outliers from the analysis. Briefly, to include samples in the study, we defined recent European ancestry (downloaded from http://csg.sph.umich.edu/chaolong/LASER/) as samples that fell into an ellipsoid spanning exclusively European population of the HGDP panel. To perform association analysis, we used SNPTEST[17] where we used frequentist additive method while adjusting for gender, and 1st twenty principal components (derived from SNP-genotypes) to correct for population structure.

UK (The INTERVAL Study) dataset

INTERVAL samples were genotyped using the Affymetrix UK Biobank Axiom array. Genotype calling was done by applying the Axiom GT1 algorithm (described in detail elsewhere[18]). Individuals who had a call rate <97% were excluded. Individuals were also excluded if contamination was indicated (>10% or >3% and >10 close relatives) and if they had a sex mismatch, or if they were not of European ancestry (PCA-based scores on PC1 or PC2<0). Individuals of non-European ancestry were identified using a set of high-quality autosomal variants and defined as variants with MAF>0.05, Hardy-Weinberg equilibrium (HWE) p value $<5 \times 10^{-6}$, and $r^2<0.2$ between pairs of variants. In the INTERVAL dataset, additional genetic markers were imputed for all autosomes using a two-step procedure. IMPUTE3 was applied for phasing of study genotypes in sets of 5000 variants with 250 variants overlapping between sets. For the imputation of variants, the Sanger Imputation Server (https://imputation.sanger.ac.uk) was used. This server implemented the Burrows-Wheeler transform imputation algorithm PBWT and analyzed whole chromosomes. A combined UK10K and the 1000 Genomes Phase 3 reference panel was used (URL, https://www.internationalgenome.org/data-portal/data-collection/phase-3). The association analysis was carried out using previously described method[19].

The UK (UK Biobank) dataset

Initially, 50,000 UK Biobank samples were genotyped using the Affymetrix UK BiLEVE Axiom array. Subsequently, the remaining 450,000 samples were genotyped using Affymetrix UK Biobank Axiom® array, which genotyped approximately 850,000 variants. There is more than 95% common content in the two arrays[20,21]. The Wellcome Trust Centre for Human Genetics performed imputation using a combination of 1000 Genomes phase 3[22], UK10K[23], and HRC[21] reference panels, and the association of all QC markers was done at deCODE using the pipeline described earlier[10].

The Netherlands (Donor InSight-III) dataset

DNA was genotyped in one batch using the UK Biobank – version 2 Axiom Array (Thermo Fisher, CA, USA), which contains 820,967 single nucleotide polymorphisms (SNP) and insertion/deletion (INDEL) markers[24]. Sample quality control (QC) by Thermo Fisher consisted of dish QC (≥0.82), QC call rate (≥97%) and copy number analyses (MAPD[2] value ≤0.35 and WavinessSD[3] value ≤0.1), and resulted in 789,754 markers[25-27]. Thermo Fisher also performed probeset QC with SNP-specific priors from the UK Biobank results using default human settings. Further QC was performed according to the UK Biobank protocol and comprised of departures from HWE calculated with bcftools, plate effects and sex effects taking into account diploid/haploid/sex specific markers. Any marker that failed at least one test ($p<10^{-6}$) was set to missing[20]. The Sanger imputation pipeline (Eagle phasing and BWT imputation using the HRC v1.1 panel) was used to impute SNPs[11,23,28]. After imputation, SNPs with an imputation score $R^2 \leq 0.3$ and minor allele frequency (MAF) ≤0.01 were

excluded. Relatedness and ethnic background were checked, and only unrelated Caucasian samples were included. Finally, principal components analysis (PCA) was carried out in PLINK2 using the autosomal genotyped SNPs and individuals that passed the QC were retained.

Meta-analysis

Briefly, we did not perform a joint meta-analysis using complete data from all cohorts, instead we used summary statistics data for RLS association from Iceland, UK Biobank, UK INTERVAL, Denmark, US Emory, and the Netherlands to run meta-analysis using fixed effect model (Mantel-Haenszel). The QC, imputation, and association tests, adjusting for principle components were done at cohort level and later meta-analyzed. Therefore, we have not done a joint principle component analysis of the discovery cohorts.

Detailed information about the QC, imputation, and the association method for Icelandic [29], UK Biobank [30], UK INTERVAL [30], and Danish (see methods) data used in this study have already been described by respective cohorts. For these samples we therefore believe that it is not necessary to generate the PCA plots. However, for the US Emory, and the Dutch Insight-III cohorts we present information in the methods section including PC plots showing genomic variation between cases and controls (below Supplementary Figure 10 and Supplementary Figure 11).

Genotyping, imputation, and association analysis of cohorts used for follow-up/replication analysis

EU-RLS-GENE consortium dataset

All samples were genotyped on the Affymetrix Axiom CEU array. Genotyping was performed following the manufacturer's protocol and implementing their best practice recommendations. Genotype calling was performed in batches based on genotyping date using the Axiom GT1 algorithm.

Individuals were excluded if they had a call rate < 98%, had incorrect or ambiguous sex calls or showed potential sample contamination. In addition, related individuals (PIHAT ≥ 0.09375) and population outliers (deviation ≥ 4 SD from the population mean in an MDS analysis) were removed. SNPs were excluded if they had a call rate < 98%, a MAF < 0.01, showed > 1 discordant genotype in duplicate samples, had a P value for deviation from HWE (P_{HWE}) ≤ 1 x 10^{-5} in controls or failed cluster quality criteria implemented in the Affymetrix SNPolisher R package. Imputation in the EU-RLS-GENE consortium GWAS was carried out in a two-step procedure. Genotype phasing was performed per chromosome using SHAPEIT2 with standard settings, followed by imputation with IMPUTE2 in all samples in chunks of 5 Mb with a buffer of 250 kb. SNPs with P_{HWE} ≤ 1 x 10^{-5} and an IMPUTE2 info score < 0.5 were filtered out prior to any statistical analysis. Finally, the association analysis was carried out using methods described elsewhere[5].

US (RBC-Omics) dataset

The RBC-omics cohort was genotyped with a custom designed Affymetrix Axiom array based off the UK Biobank Array, which included probes for ~879,000 SNPs [7]. All genotype calls and quality control steps were performed with the Affymetrix Power Tools according to their best practice workflow. Individuals with <97% call rate or Dish QC (DQC) < 0.85 were excluded from all subsequent analysis, as were individuals with sex mismatch. Genotypes were called using two different batch groupings, and SNPs with inconsistent calls in five or more individuals were excluded from analysis. Low and medium confidence called SNPs were also excluded, resulting in 831,797 high confidence SNPs. Cryptic relatedness among individuals was determined via PLINK identity-by-descent analysis. For pairs of individuals having PI_HAT > 0.4 or IBS > 0.9 the member with fewer SNP calls was excluded from analysis. Finally, SNPs deviating from HWE ($P \leq 1 \times 10^{-4}$) were also excluded. The resulting 811,784 SNPs for 12,789 individuals were used to perform statistical phasing with SHAPEIT and imputation of unmeasured genotypes with IMPUTE2 using a cosmopolitan sample of 1000 Genome Project phase 3 for reference haplotypes. Caucasian ancestry was determined using STRUCTURE and individuals were compared to 1000 Genome reference populations. Individuals who self-reported as Caucasian and were >80% European Ancestry were used in the analysis. The final high-quality dataset had 8.1 million SNPs at 5% MAF and 14 million SNPs at 1% MAF. Alleles at MAF < 1% were excluded. Association of the five variants identified in the discovery meta-analysis with RLS, was investigated using a multivariate logistic regression model adjusted for sex, age and the first 10 principal component scores.

Supplementary Figure 1. The regional association plots 'LocusZoom' for lead variant 'rs10188680-T'

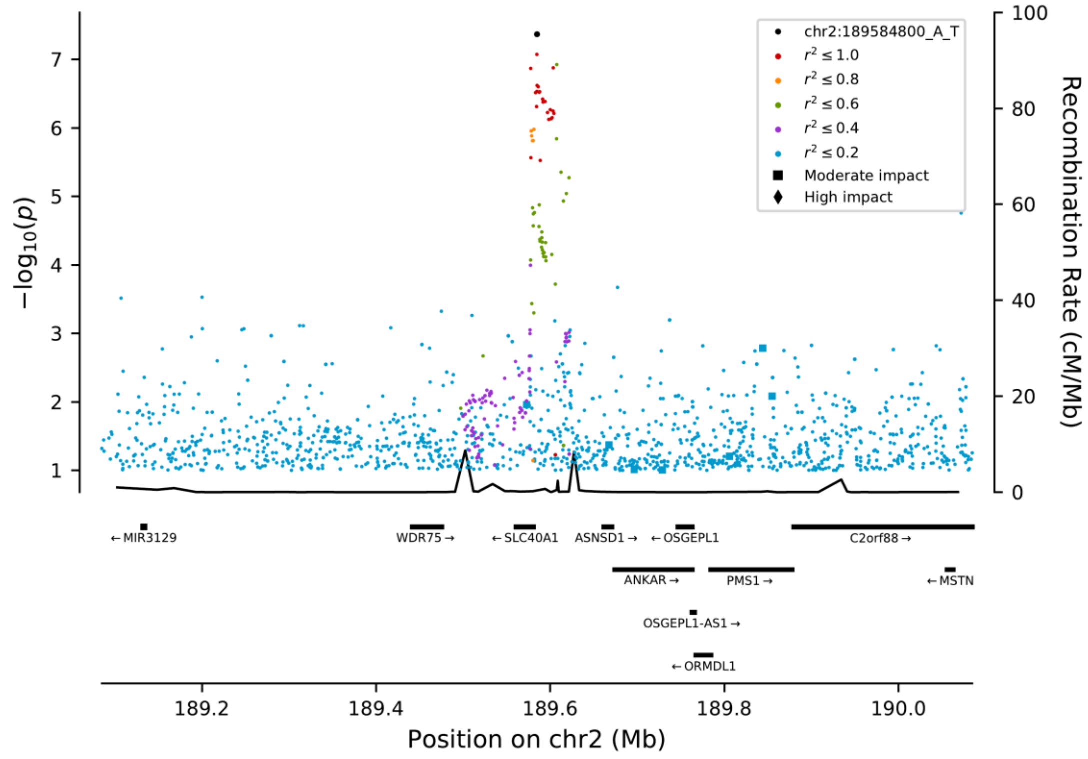

Supplementary Figure 1: The regional association plots 'LocusZoom' for lead variant 'rs10188680-T' association (flanked by ±500KB) with RLS in the discovery meta-analysis. Each dot represents its association strength '-log10(*P*)' with RLS. The color code represents ,r2, measure the LD of the lead variant with surrounding variants.

Supplementary Figure 2. The regional association plots 'LocusZoom' for lead variant 'rs10068599-T'

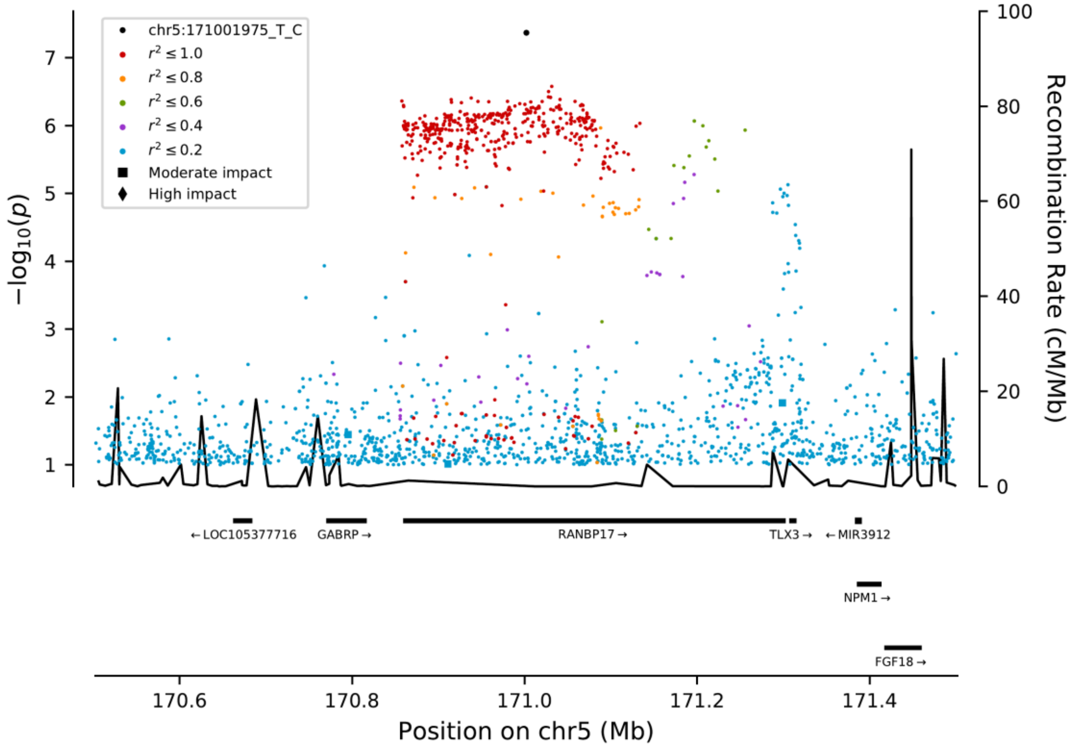

Supplementary Figure 2: The regional association plots 'LocusZoom' for lead variant 'rs10068599-T' association (flanked by ±500KB) with RLS in the discovery meta-analysis. Each dot represents its association strength '-log10(*P*)' with RLS. The color code represents ,r2, measure the LD of the lead variant with surrounding variants.

Supplementary Figure 3. The regional association plots 'LocusZoom' for lead variant 'rs112716420-G'

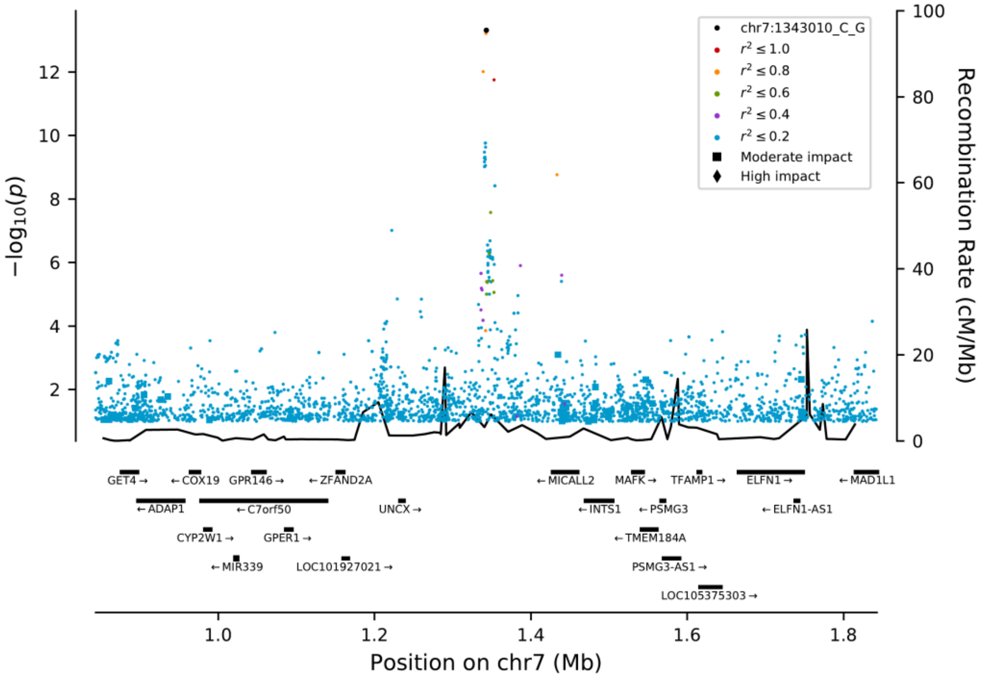

Supplementary Figure 3: The regional association plots 'LocusZoom' for lead variant 'rs112716420-G' association (flanked by ±500KB) with RLS in the discovery meta-analysis. Each dot represents its association strength '-log10(*P*)' with RLS. The color code represents ,r2, measure the LD of the lead variant with surrounding variants.

Supplementary Figure 4. The regional association plots 'LocusZoom' for lead variant 'rs10769894-A'

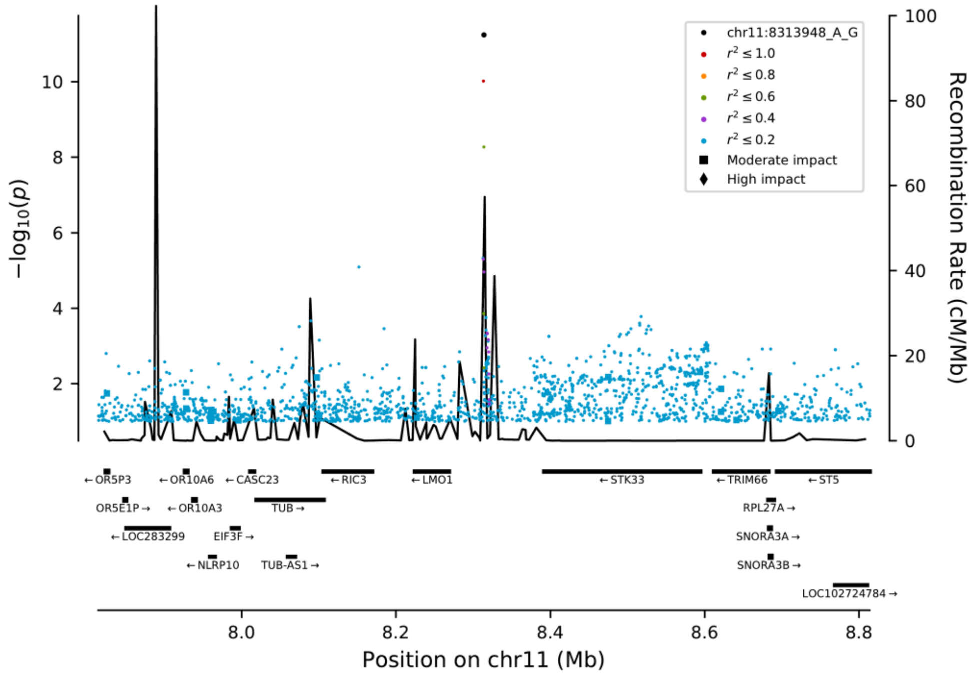

Supplementary Figure 4: The regional association plots 'LocusZoom' for lead variant 'rs10769894-A' association (flanked by ±500KB) with RLS in the discovery meta-analysis. Each dot represents its association strength '-log10(*P*)' with RLS. The color code represents ,r2, measure the LD of the lead variant with surrounding variants.

Supplementary Figure 5. The regional association plots 'LocusZoom' for lead variant 'rs58127855-T'

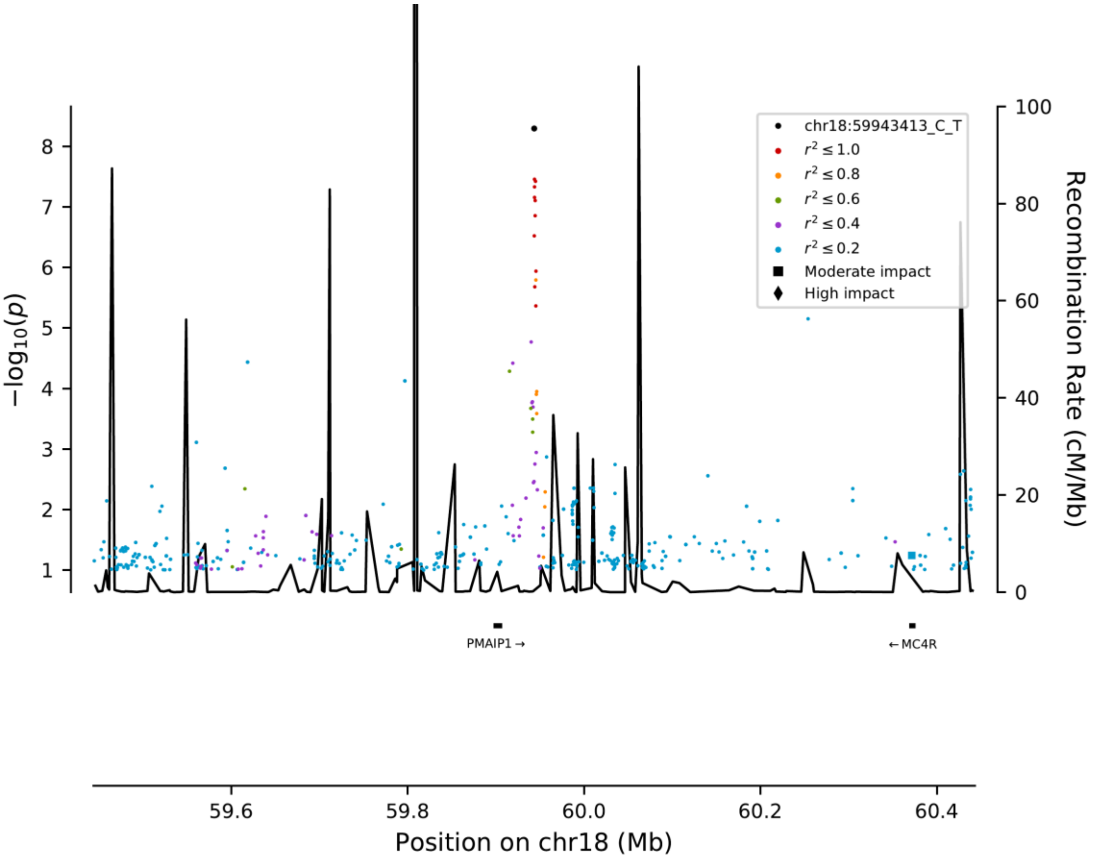

Supplementary Figure 5: The regional association plots 'LocusZoom' for lead variant 'rs58127855-T' association (flanked by ±500KB) with RLS in the discovery meta-analysis. Each dot represents its association strength '-log10(*P*)' with RLS. The color code represents ,r2, measure the LD of the lead variant with surrounding variants.

Supplementary Figure 6. Cis gene expression analysis of RLS variants using 54 GTEx tissues

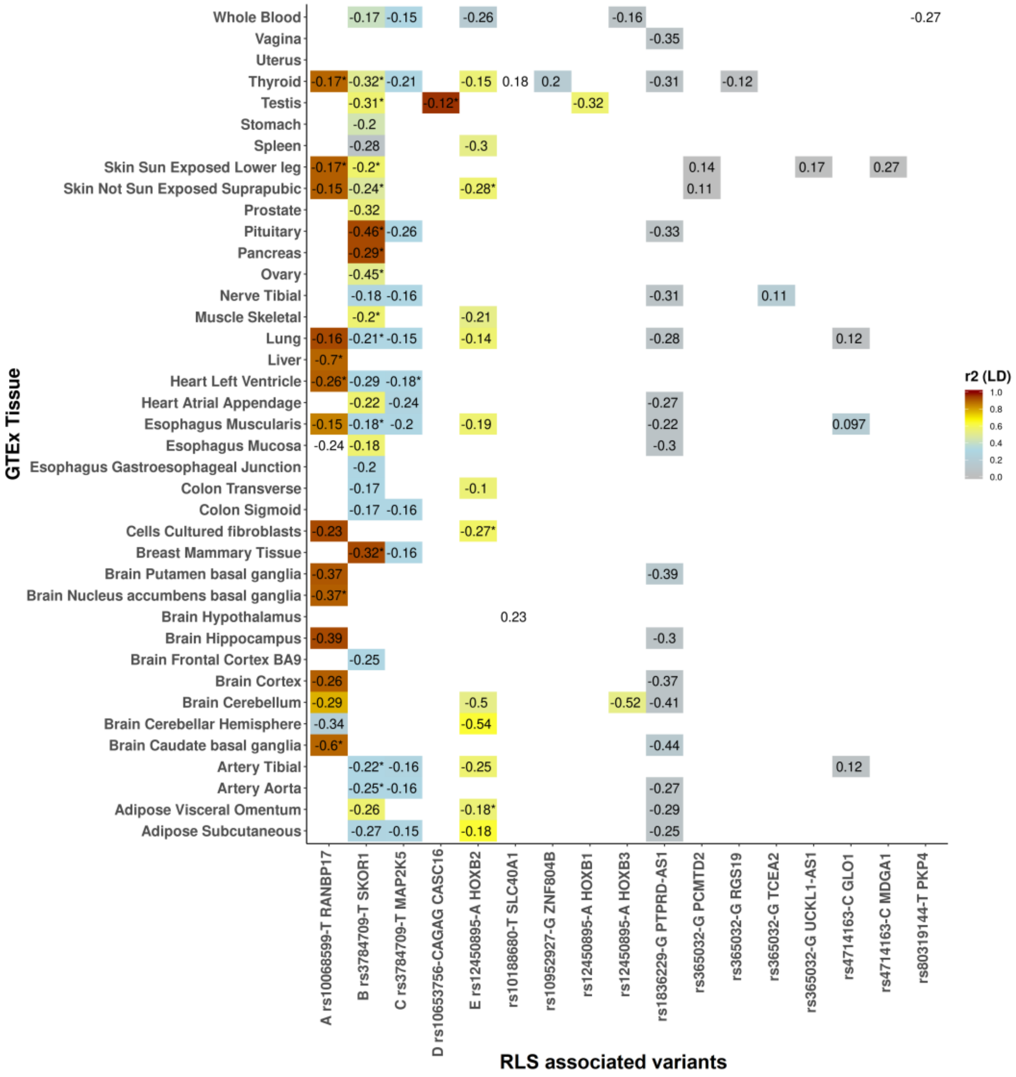

Supplementary figure 6: Of the 23 RLS variants, we found cis-eQTL data for 11 of the 23 RLS variants impacting 17 genes. Four variants are significantly associated with cis gene expression at least in one tissue tested and are in high linkage disequilibrium (LD) (r2>0.90) with the top eQTL variant of each respective gene. These are rs10068599-T lowering the expression of *RANBP17* in the tissues thyroid, sun exposed skin on lower leg, liver, the left ventricle of the heart, and brain tissues in the basal ganglia. Moreover, the rs3784709-T

which lowers the expression of *SKOR1 and MAP2K5* in tissues pituitary, pancreas, and breast mammary tissue. Finally, the rs10653756-CACAG lowering the expression of *CASC16* in testis. Cis-eQTL effect estimates (normalized) are provided and those sharing same causal signal (COJO conditional analysis) with eQTL and are Bonferroni significant ($P < 3.3 \times 10^{-06}$) are labeled with an asterisk.

Genetic risk analyses: UK Biobank

Analyses where the polygenic risk score for RLS was applied as the exposure variable were conducted among individuals included in the UK Biobank cohort. UK Biobank is an open access research resource with data on 500,000 UK adults (ages 40 years and up). Genotyping was carried out on all participants and several baseline measurements were conducted, including a comprehensive self-report health related questionnaire that was administered to all included individuals, which among other things contained questions regarding diet, cognitive functioning, work history and digestive health. Moreover, the UK Biobank has linked to individual level data from hospitals and general practitioners.

Supplementary figure 7. The phenotypic variance explained by the RLS polygenic risk score (PRS) using different P-parameters from the Linkage Disequilibrium Score regression (LDSC) method

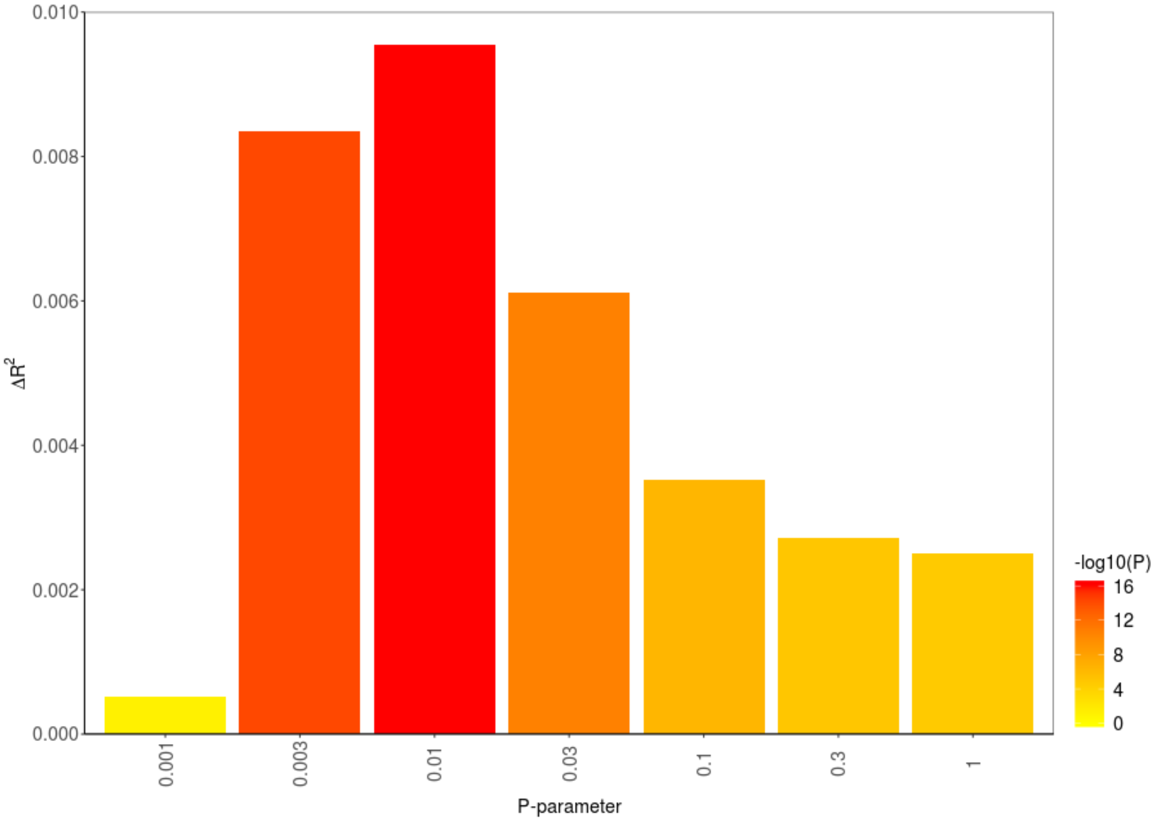

Supplementary figure 7: RLS PRS for UKB Biobank participants was constructed using GWAS meta-analysis of Iceland, Denmark, US Emory, and the Netherlands. Therein, the largest variance (0.97%) is explained by the '0.01' threshold for ICD 10 G25.8 in UK Biobank. This PRS was calculated in N = 12,075 independent biological samples and using a threshold of 0.01 it was further used to perform phenome-wide PRS association analysis of RLS PRS in UK Biobank for case-control (disease/phenotypes) and quantitative traits. The number of variants included in this RLS-PRS is 600,420.

Supplementary Figure 8. ROC curve showing sensitivity and specificity of RLS PRS vs corresponding ICD 10-code G25.8 phenotype in the UK Biobank

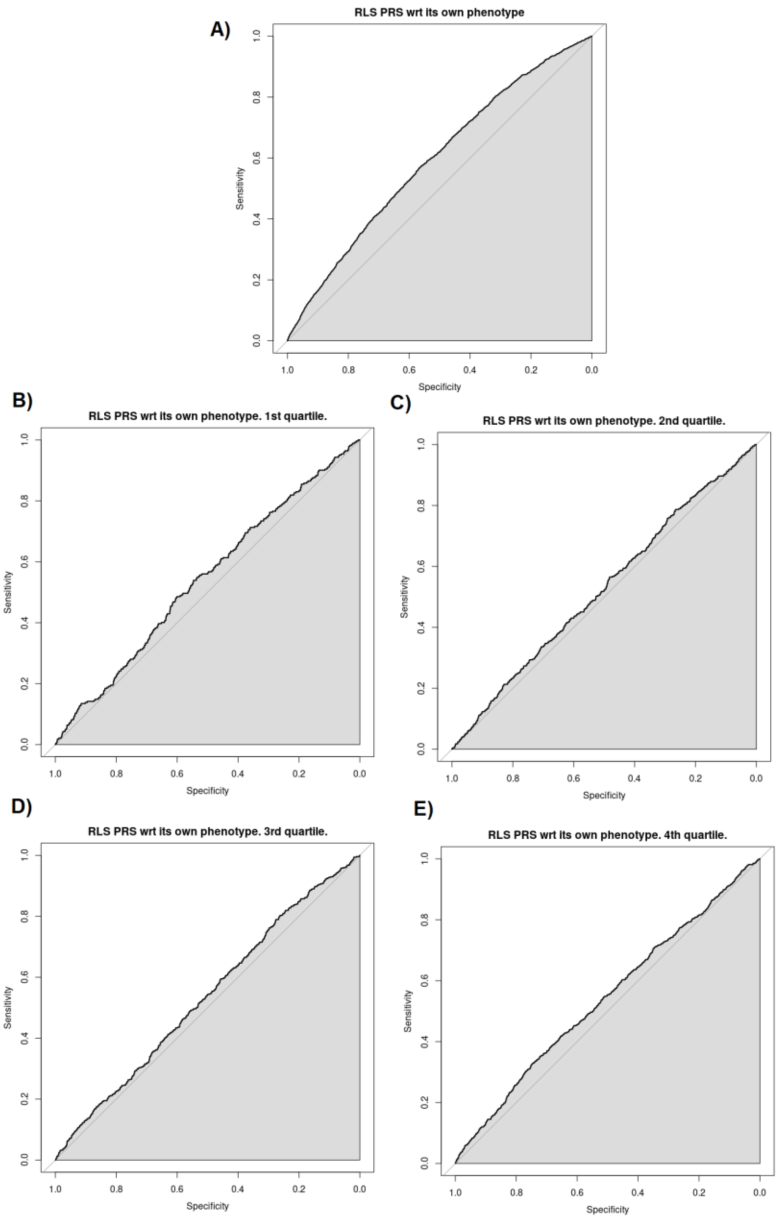

Supplementary Figure 8**:** ROC curve showing sensitivity and specificity of RLS PRS vs corresponding ICD 10-code G25.8 phenotype in the UK Biobank, where **A**) is for all and B-E) are for 1st, 2nd, 3rd and 4th quartile respectively. Analyses were done for N = 12,075 independent biological samples.

Supplementary figure 9. QQ-plots for discovery association analysis

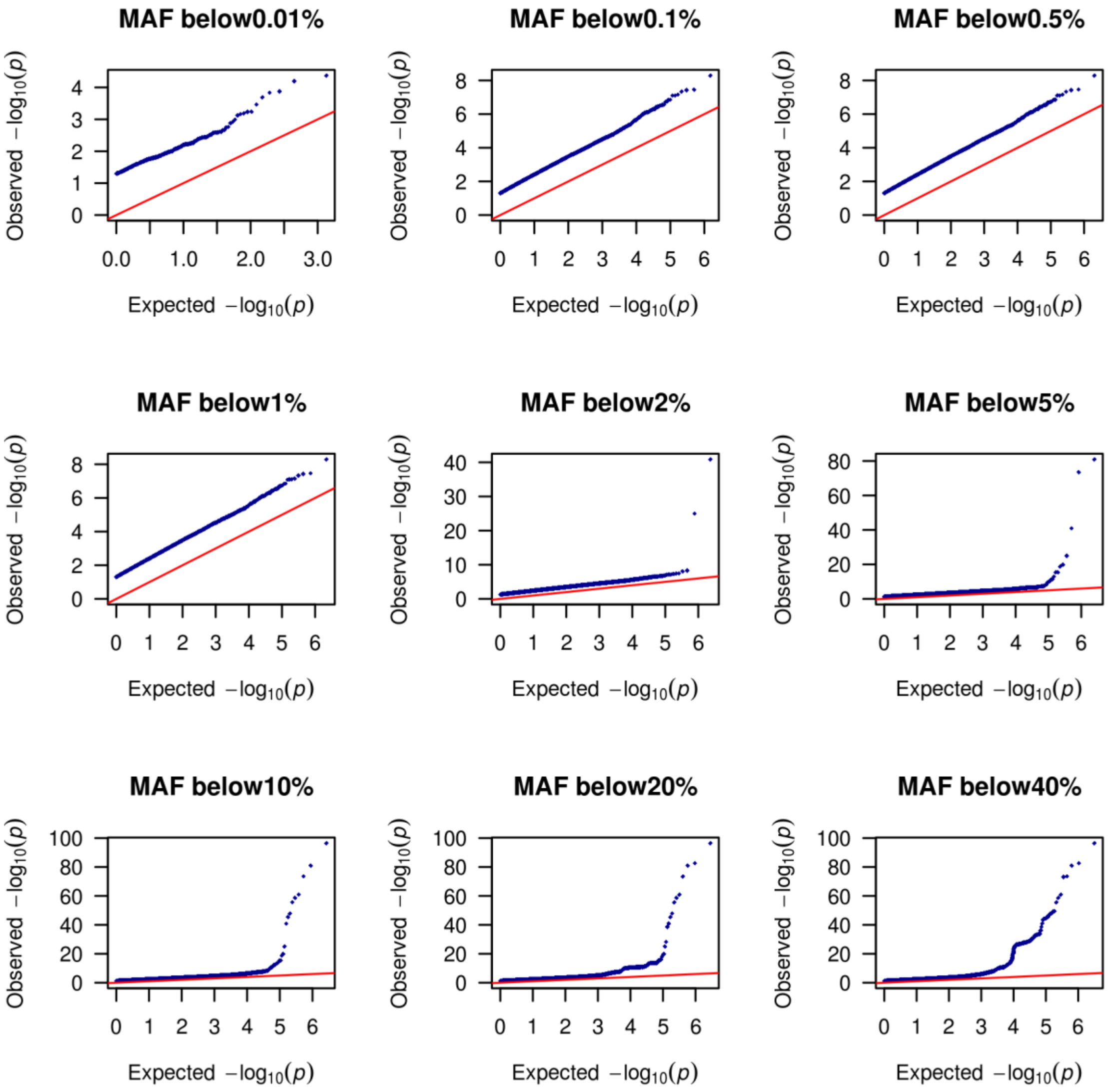

Supplementary figure 9: QQ-plot of P values for RLS association results. Expected versus observed P values. Therein, we present minor allele frequency (MAF) based split plots, for detailed description of observed P distribution versus expected P.

Supplementary figure 10. Principal component analysis plot for the Netherlands cohort

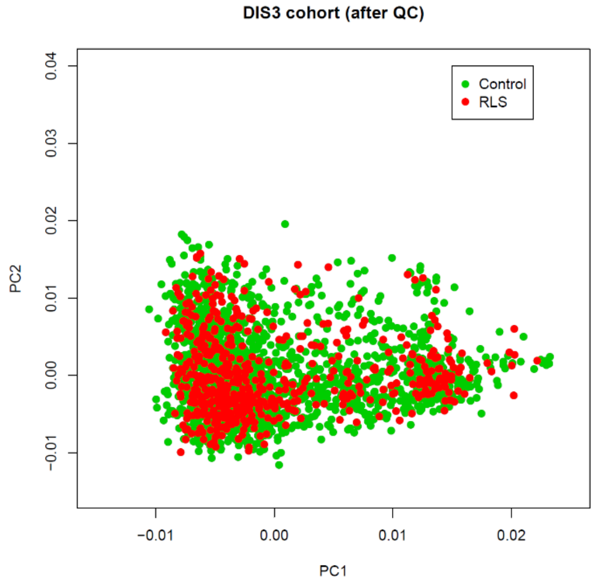

Supplementary figure 10: Principal component analysis plot for the Netherlands cohort with N = 2,363 independent biological samples (565 RLS cases and 1,798 controls). On the x-axis is PC1 and on y-axis PC2. Each dot represents participant in the study from Dutch cohort where RLS cases are red and controls in green color. The plot shows that most of the variation in cases and controls is captured through PC1 & PC2. In the association analysis, to be conservative we used 1st 10 PC to correct for variations arising due to population structure.

Supplementary figure 11. Principal component analysis (PCA) plot for US Emory cohort

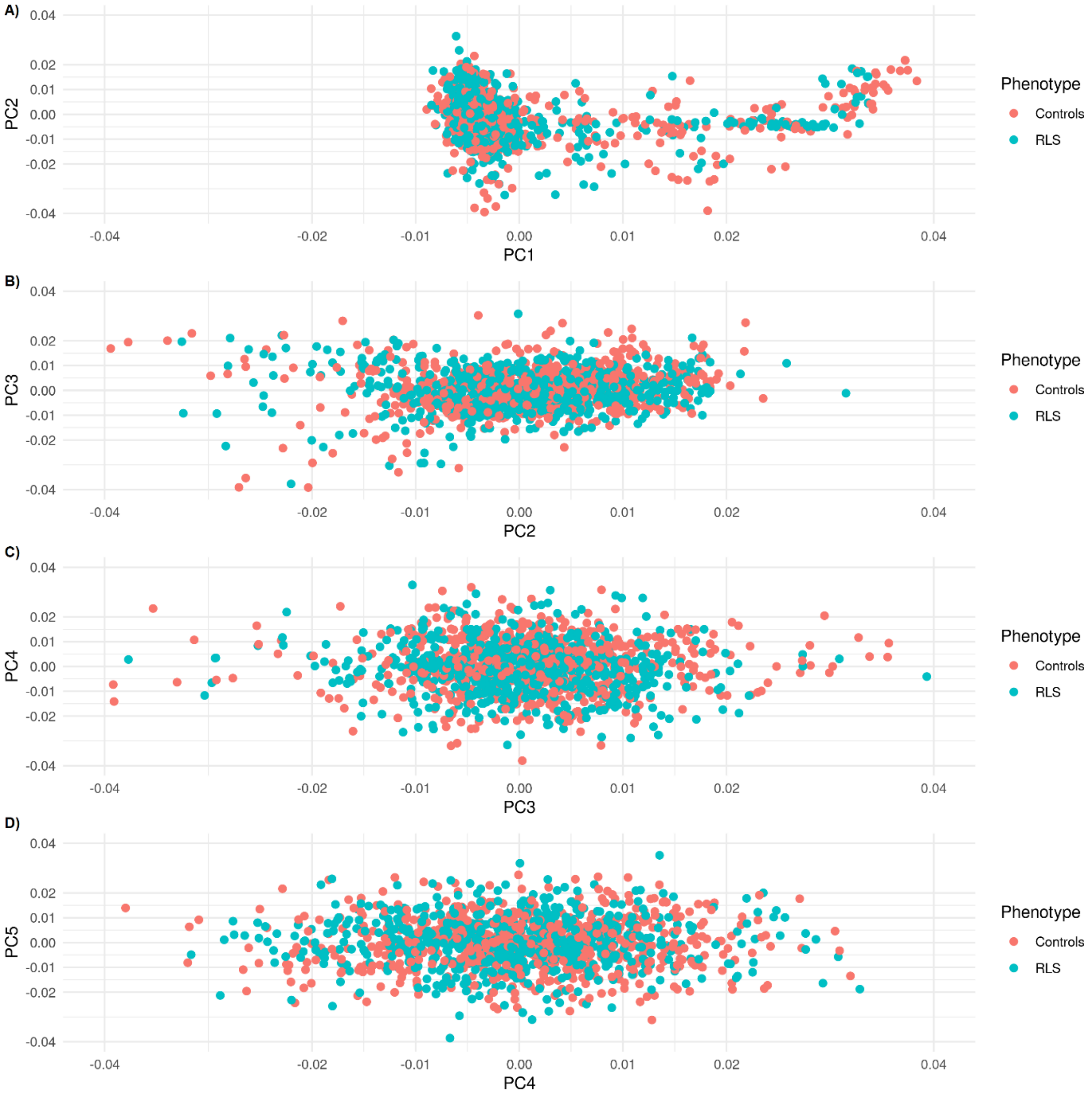

Supplementary figure 11: Principal component analysis (PCA) plot for US Emory cohort with N = 1,417 independent biological sample (696 RLS cases and 721 controls). On the x-axis **A)** through **D)** is PC1, PC2, PC3, and PC4. Whereas on y-axis **A)** through **D) is** PC2, PC3, PC4, and PC5. Each dot represents the participant in the study from US Emory cohort

where RLS cases are colored green and controls in red color. These plots show that most of the variation in cases and controls is captured through PC1 – PC5. Furthermore, in the association analysis, we used 1st 20 PC to correct for variations arising due to population structure.

Supplementary references

1 Allen, R. P. *et al.* Restless legs syndrome/Willis-Ekbom disease diagnostic criteria: updated International Restless Legs Syndrome Study Group (IRLSSG) consensus criteria--history, rationale, description, and significance. *Sleep Med* **15**, 860-873, doi:10.1016/j.sleep.2014.03.025 (2014).

2 Allen, Burchell, MacDonald, Hening & Earley. Validation of the self-completed Cambridge-Hopkins questionnaire (CH-RLSq) for ascertainment of restless legs syndrome (RLS) in a population survey. *Sleep Med* **10**, 1097-1100, doi:10.1016/j.sleep.2008.10.007 (2009).

3 Timmer, T. C. *et al.* Donor InSight: characteristics and representativeness of a Dutch cohort study on blood and plasma donors. *Vox sanguinis* **114**, 117-128, doi:10.1111/vox.12731 (2019).

4 Spencer, B. R. *et al.* Restless legs syndrome, pica, and iron status in blood donors. *Transfusion* **53**, 1645-1652, doi:10.1111/trf.12260 (2013).

5 Schormair, B. *et al.* Identification of novel risk loci for restless legs syndrome in genome-wide association studies in individuals of European ancestry: a meta-analysis. *The Lancet. Neurology* **16**, 898-907, doi:10.1016/s1474-4422(17)30327-7 (2017).

6 Endres-Dighe, S. M. *et al.* Blood, sweat, and tears: Red Blood Cell-Omics study objectives, design, and recruitment activities. *Transfusion*, doi:10.1111/trf.14971 (2018).

7 Yuelong Guo, M. B., Mark Seielstad, Stacy Endres-Dighe, Connie M. Westhoff, Brendan Keating, Carolyn Hoppe, Aarash Bordbar, Brian Custer, Adam S. Butterworth, Tamir Kanias, Alan E. Mast, Steve Kleinman, Yontao Lu, and Grier P. Page. Development and Evaluation of a Transfusion Medicine Genome Wide Genotyping Array. *Transfusion* **in press** (2018).

8 Kanias, T. *et al.* Ethnicity, sex, and age are determinants of red blood cell storage and stress hemolysis: results of the REDS-III RBC-Omics study. *Blood advances* **1**, 1132-1141, doi:10.1182/bloodadvances.2017004820 (2017).

9 Kong, A. *et al.* Detection of sharing by descent, long-range phasing and haplotype imputation. *Nature genetics* **40**, 1068-1075, doi:10.1038/ng.216 (2008).

10 Steinthorsdottir, V. *et al.* Common variants upstream of KDR encoding VEGFR2 and in TTC39B associate with endometriosis. *Nature communications* **7** (2016).

11 Loh, P. R. *et al.* Reference-based phasing using the Haplotype Reference Consortium panel. *Nature genetics* **48**, 1443-1448, doi:10.1038/ng.3679 (2016).

12 Jonsson, H. *et al.* Whole genome characterization of sequence diversity of 15,220 Icelanders. *Scientific data* **4**, 170115, doi:10.1038/sdata.2017.115 (2017).

13 Delaneau, O., Howie, B., Cox, A. J., Zagury, J. F. & Marchini, J. Haplotype estimation using sequencing reads. *American journal of human genetics* **93**, 687-696, doi:10.1016/j.ajhg.2013.09.002 (2013).

14 Howie, B. N., Donnelly, P. & Marchini, J. A flexible and accurate genotype imputation method for the next generation of genome-wide association studies. *PLoS genetics* **5**, e1000529, doi:10.1371/journal.pgen.1000529 (2009).

15 Alexander, D. H., Novembre, J. & Lange, K. Fast model-based estimation of ancestry in unrelated individuals. *Genome Res* **19**, 1655-1664, doi:10.1101/gr.094052.109 (2009).

16 Price, A. L. *et al.* Principal components analysis corrects for stratification in genome-wide association studies. *Nature genetics* **38**, 904-909, doi:10.1038/ng1847 (2006).

17 Marchini, J., Howie, B., Myers, S., McVean, G. & Donnelly, P. A new multipoint method for genome-wide association studies by imputation of genotypes. *Nature genetics* **39**, 906-913, doi:10.1038/ng2088 (2007).

18 Di Angelantonio, E. *et al.* Efficiency and safety of varying the frequency of whole blood donation (INTERVAL): a randomised trial of 45 000 donors. *Lancet (London, England)* **390**, 2360-2371, doi:10.1016/s0140-6736(17)31928-1 (2017).

19 Ji, S. G. *et al.* Genome-wide association study of primary sclerosing cholangitis identifies new risk loci and quantifies the genetic relationship with inflammatory bowel disease. *Nature genetics* **49**, 269-273, doi:10.1038/ng.3745 (2017).

20 Bycroft, C. *et al.* The UK Biobank resource with deep phenotyping and genomic data. *Nature* **562**, 203-209, doi:10.1038/s41586-018-0579-z (2018).

21 Bycroft, C. *et al.* Genome-wide genetic data on~ 500,000 UK Biobank participants. 166298 (2017).

22 Auton, A. *et al.* A global reference for human genetic variation. *Nature* **526**, 68-74, doi:10.1038/nature15393 (2015).

23 McCarthy, S. *et al.* A reference panel of 64,976 haplotypes for genotype imputation. *Nature genetics* **48**, 1279-1283, doi:10.1038/ng.3643 (2016).

24 Biobank., U. https://www.ukbiobank.ac.uk/scientists-3/genetic-data/.

25 Affymetrix, I. (URL, 2013).

26 Affymetrix, l. UKB_WCSGAX: UK Biobank 500K Samples Processing by the Affymetrix1 Research Services Laboratory. *URL* (2017).

27 Affymetrix, I. UKB_WCSGAX: UK Biobank 500K Samples Genotyping Data Generation by the Affymetrix Research Services Laboratory. *URL* (2015).

28 Durbin, R. Efficient haplotype matching and storage using the positional Burrows-Wheeler transform (PBWT). *Bioinformatics (Oxford, England)* **30**, 1266-1272, doi:10.1093/bioinformatics/btu014 (2014).

29 Styrkarsdottir, U. *et al.* GWAS of bone size yields twelve loci that also affect height, BMD, osteoarthritis or fractures. *Nature communications* **10**, 1-13 (2019).

30 Astle, W. J. *et al.* The allelic landscape of human blood cell trait variation and links to common complex disease. *Cell* **167**, 1415-1429. e1419 (2016).
