# Supplementary material for: Large genome-wide association study identifies three novel risk variants for restless legs syndrome
Source: Commun Biol. 2020 Nov 25;3:703. doi: 10.1038/s42003-020-01430-1 (PMC7689502; doi:10.1038/s42003-020-01430-1)
Supplement: Supplementary file 3 — Description of Additional Supplementary Files [file 42003_2020_1430_MOESM3_ESM.pdf]

Description of Additional Supplementary Files

File Name: Supplementary Data 1 – 12

Description:

• **Supplementary Data 1**: Displaying association results for the five novel RLS-associated variants identified in the discovery meta-analysis

• **Supplementary Data 2**: Displaying association results for previously identified RLS-associated variants as well as association results for these variants in the discovery cohort from the present study as well as a meta-analysis of the results from the discovery cohort and previous results

• **Supplementary Data 3**: Displaying novel RLS-associated variants identified in the discovery meta-analysis as well as associations with known RLS-associated variants

• **Supplementary Data 4**: Displaying genotype tissue expression for RLS-associated variants

• **Supplementary Data 5**: Displaying results from COJO conditional analysis of GTEx expression data for all variants with prior probablity of P value < 3.3e-6 and degree of linkage disequilibrium>=0.3 with the lead RLS-associated variants. analysis and therefore set Bonferroni threshold to P value < 2.01e-04

• **Supplementary Data 6**: COJO conditional analysis of GTEx expression data for all variants with prior probability of P < 3.3e-6 and r2>=0.3 with the RLS lead variants. We performed 249 tests (220+29) for COJO conditional analysis and therefore set the Bonferroni threshold to P < 2.01e-04 for different signals.

• **Supplementary Data 7**: Displaying AUC calculation for RLS cases in UK Biobank using: phenotype ~ rls_prs + Sex*poly(Yob,3) + PC1:40.

• **Supplementary Data 8**: Displaying results from association analysis between RLS-polygenic risk score and several binary health-related traits

• **Supplementary Data 9**: Displaying results from association analysis between RLS-polygenic risk score and several quantitative health-related traits

• **Supplementary Data 10**: Displaying measures of genetic correlations using LDSC gc method for the top ranked binary traits from the RLS-polygenic risk score analysis

• **Supplementary Data 11**: Displaying measure of genetic correlation using LDSC gc method for the top ranked quantitative traits from PRS analysis.

• **Supplementary Data 12**: Displaying top gene-set terms/pathways from MAGMA gene-set enrichment analysis using Molecular signature database resource.
