# Supplementary material for: Large genome-wide association study identifies three novel risk variants for restless legs syndrome
Source: Commun Biol. 2020 Nov 25;3:703. doi: 10.1038/s42003-020-01430-1 (PMC7689502; doi:10.1038/s42003-020-01430-1)
Supplement: Supplementary file 5 — Reporting Summary [file 42003_2020_1430_MOESM5_ESM.pdf]

nature research

Corresponding author(s): Henrik Ullum

Last updated by author(s): Oct 9, 2020

Reporting Summary

Nature Research wishes to improve the reproducibility of the work that we publish. This form provides structure for consistency and transparency in reporting. For further information on Nature Research policies, see our Editorial Policies and the Editorial Policy Checklist.

Statistics

For all statistical analyses, confirm that the following items are present in the figure legend, table legend, main text, or Methods section.

n/a	Confirmed	
☐	☒	The exact sample size (*n*) for each experimental group/condition, given as a discrete number and unit of measurement
☒	☐	A statement on whether measurements were taken from distinct samples or whether the same sample was measured repeatedly
☐	☒	The statistical test(s) used AND whether they are one- or two-sided<br>*Only common tests should be described solely by name; describe more complex techniques in the Methods section.*
☐	☒	A description of all covariates tested
☐	☒	A description of any assumptions or corrections, such as tests of normality and adjustment for multiple comparisons
☐	☒	A full description of the statistical parameters including central tendency (e.g. means) or other basic estimates (e.g. regression coefficient) AND variation (e.g. standard deviation) or associated estimates of uncertainty (e.g. confidence intervals)
☐	☒	For null hypothesis testing, the test statistic (e.g. *F*, *t*, *r*) with confidence intervals, effect sizes, degrees of freedom and *P* value noted<br>*Give P values as exact values whenever suitable.*
☒	☐	For Bayesian analysis, information on the choice of priors and Markov chain Monte Carlo settings
☒	☐	For hierarchical and complex designs, identification of the appropriate level for tests and full reporting of outcomes
☐	☒	Estimates of effect sizes (e.g. Cohen's *d*, Pearson's *r*), indicating how they were calculated

Our web collection on statistics for biologists contains articles on many of the points above.

Software and code

Policy information about availability of computer code

Data collection	We used publicly available software (URLs listed below) in conjunction with the sequencing processing pipeline:<br><br>GTEx, https://www.gtexportal.org/. The Genotype-Tissue Expression (GTEx).<br>COJO, https://cnsgenomics.com/software/gcta/#Overview.<br>SHAPEIT, https://mathgen.stats.ox.ac.uk/genetics_software/shapeit/shapeit.html.<br>PLINK2, https://www.cog-genomics.org/plink/2.0/<br>IMPUTE 2, https://mathgen.stats.ox.ac.uk/impute/impute_v2.html#download<br><br>We used R extensively to analyze data and create plots.
Data analysis	We used publicly available software (URLs listed below) in conjunction with algorithms in the sequencing processing pipeline:<br><br>GTEx, https://www.gtexportal.org/. The Genotype-Tissue Expression (GTEx).<br>COJO, https://cnsgenomics.com/software/gcta/#Overview.<br>SHAPEIT, https://mathgen.stats.ox.ac.uk/genetics_software/shapeit/shapeit.html.<br>PLINK2, https://www.cog-genomics.org/plink/2.0/<br>IMPUTE 2, https://mathgen.stats.ox.ac.uk/impute/impute_v2.html#download<br><br>We used R extensively to analyze data and create plots.

For manuscripts utilizing custom algorithms or software that are central to the research but not yet described in published literature, software must be made available to editors and reviewers. We strongly encourage code deposition in a community repository (e.g. GitHub). See the Nature Research guidelines for submitting code & software for further information.

Data

Policy information about availability of data

All manuscripts must include a data availability statement. This statement should provide the following information, where applicable:

- Accession codes, unique identifiers, or web links for publicly available datasets
- A list of figures that have associated raw data
- A description of any restrictions on data availability

The RLS meta-analysis summary statistics will be made available at https://www.decode.com/summarydata/.
For information on further access to data included in the meta-analysis, please contact the following authors in charge of the respective cohorts:
Henrik Ullum for data from the Danish Blood Donor Study (henrik.ullum@regionh.dk), Hreinn Stefansson for data from the Icelandic cohort (hreinn.stefansson@decode.is), David B. Rye for data from the Emory cohort (rlsrye@gmail.com), Emanuele Di Angelantonio for the INTERVAL cohort (ed303@medschl.cam.ac.uk), and Katja Van Den Hurk for data from the Donor Insight-III (k.vandenhurk@sanquin.nl). For UK Biobank please register on https://bbams.ndph.ox.ac.uk/ams/ and apply for the data through there.

Field-specific reporting

Please select the one below that is the best fit for your research. If you are not sure, read the appropriate sections before making your selection.

☒ Life sciences ☐ Behavioural & social sciences ☐ Ecological, evolutionary & environmental sciences

For a reference copy of the document with all sections, see nature.com/documents/nr-reporting-summary-flat.pdf

Life sciences study design

All studies must disclose on these points even when the disclosure is negative.

Sample size	The GWAS meta-analysis included 480,982 (10,257 cases and 470,725 controls) adults of European ancestry.
Data exclusions	With the exception of standard, pre-established data quality control procedures specified in the Methods section, no data was excluded.
Replication	Novel variants identified in the discovery phase of our study were tested for association in two replication datasets consisting of subjects of European ancestry, the EU-RLS-GENE consortium6 (6,228 cases and 10,992 controls) and the RBC-Omics cohort (423 cases and 7,334 controls).
Randomization	No randomization was used.
Blinding	Not relevant for this study, as this is a GWAS meta-analysis study.

Reporting for specific materials, systems and methods

We require information from authors about some types of materials, experimental systems and methods used in many studies. Here, indicate whether each material, system or method listed is relevant to your study. If you are not sure if a list item applies to your research, read the appropriate section before selecting a response.

Materials & experimental systems

n/a	Involved in the study	
☒	☐	Antibodies
☒	☐	Eukaryotic cell lines
☒	☐	Palaeontology and archaeology
☒	☐	Animals and other organisms
☒	☐	Human research participants
☒	☐	Clinical data
☒	☐	Dual use research of concern

Methods

n/a	Involved in the study	
☒	☐	ChIP-seq
☒	☐	Flow cytometry
☒	☐	MRI-based neuroimaging
